# Bringing together conceptualisations of the health advocacy competence across the continuum of medical education: a scoping review protocol

**DOI:** 10.1136/bmjopen-2024-097894

**Published:** 2025-07-25

**Authors:** Wilma R W Oosthoek, Dario Cecilio-Fernandes, Maarten F M Engel, Lars T van Prooijen, Suzie J Otto, Andrea M Woltman

**Affiliations:** 1 Institute for Medical Education Research Rotterdam, Erasmus MC University Medical Center Rotterdam, Rotterdam, The Netherlands; 2 Department of Research and Education, Medical Library, Erasmus MC University Medical Center Rotterdam, Rotterdam, The Netherlands; 3 Department of Public Health, Erasmus MC University Medical Center Rotterdam, Rotterdam, The Netherlands

**Keywords:** Education, Medical, Clinical Competence, Review

## Abstract

**Introduction:**

Health advocacy (HA) is acknowledged as a core competence in medical education. However, varying and sometimes conflicting conceptualisations of HA exist, making it challenging to integrate the competence consistently. While this diversity highlights the need for a deeper understanding of HA conceptualisations, a comprehensive analysis across the continuum of medical education is absent in the literature. This protocol has been developed to clarify the conceptual dimensions of the HA competence in literature as applied to medical education.

**Methods and analysis:**

The review will be conducted in line with the JBI (formerly Joanna Briggs Institute) methodology for scoping reviews. A comprehensive literature search was developed and already carried out in eight academic databases and Google Scholar, without restrictions on publication date, geography or language. Articles that describe the HA role among students and physicians who receive or provide medical education will be eligible for inclusion. Two independent reviewers will independently complete title and abstract screening prior to full-text review of selected articles and data extraction on the final set. A descriptive-analytical approach will be applied for summarising the data.

**Ethics and dissemination:**

This scoping review does not involve human participants, as all evidence is sourced from publicly available databases. Therefore, ethical approval is not required for this study. The findings from this scoping review will be disseminated through submission to a high-quality peer-reviewed journal and presented at academic conferences. By clarifying the conceptualisations of HA, this review aims to contribute to a shared narrative that will strengthen the foundation for integrating the HA role into medical education.

**Trial registration number:**

A preliminary version of this protocol was registered on the Open Science Framework on 9 December 2024, and can be accessed at the following link: https://osf.io/ed2br. We have also registered our scoping review protocol as a preprint at medRxiv: https://doi.org/10.1101/2024.12.09.24318699.

**STRENGTHS AND LIMITATIONS OF THIS STUDY**

- This review will maintain consistency and meet the quality standards established by other scoping reviews by adhering to the JBI (formerly Joanna Briggs Institute) methodology for scoping reviews and following the Preferred Reporting Items for Systematic Reviews and Meta-Analyses extension for Scoping Reviews checklist.
- Two independent reviewers will conduct the title and abstract screening, full-text screening and data extraction, including pilot exercises, to enhance the systematic rigour of the screening process and increase the reliability of the results.
- The search strategy is systematically developed in collaboration with a medical librarian, who also serves as a coauthor on this project.
- This review ensures inclusivity by avoiding language restrictions, designing data visualisations for colour-blind readers and committing to open-access resources to align with FAIR (findable, accessible, interoperable and reusable) principles.
- This review scopes the whole field of medical education, as defined following the definition of the World Medical Association.

## Introduction

 Health advocacy (HA) is a core competence for both current and future medical professionals to shape the healthcare system into one that is inclusive, accessible, affordable and sustainable for both individuals and populations.^1 2^ Although the Royal College of Physicians and Surgeons of Canada introduced the concept of HA into medical education through the CanMEDS framework,^3^ several other concepts have since emerged in the literature, making consistent integration of the competence challenging. Previous reviews on HA in medical education have addressed teaching and assessment methods related to HA at specific stages of medical education,^4^,^8^ but less attention has been given to the emerging variety of HA conceptualisations. To bridge this gap, there is a need for a comprehensive analysis that brings clarity to the variety of HA conceptualisations across the continuum of medical education.

CanMEDS defines HA competence as a role where physicians work with individuals, communities and populations to improve health outcomes, advocate for those they serve and mobilise resources for change.^9^ However, this definition has been expanded, refined or challenged by others, incorporating various perspectives to address ambiguities around the role.^10^ HA is viewed as a clinical agent’s role, focusing on health promotion, compassionate patient-centred care and/or access to resources on an individual level^11^,^14^; as an activist role, addressing health injustices at both local and global levels^11^,^1315 16^; as a personal role shaped by individual values and beliefs that drive perceptions and behaviours^17^,^19^ and as a professional role influenced by the social contract between the medical profession and society, as well as by societal norms and values.^18 20^ Due to its diverse interpretations, overlap with other competencies and fluid, unclear boundaries, the HA role is one of the least understood and assessed in medical education.^10 21^

The wide range of conceptualisations allows medical students and physicians to align their advocacy efforts with their personal values, providing them with a sense of purpose and fulfilment.^17 22^ While the flexibility of the role offers opportunities for change, it also generates ethical tensions. The conceptual boundaries between the HA role and medical expert role as defined and described by CanMEDS are often blurred, contributing to conflicting views on how far medical professionals should go in promoting societal or systemic reform as part of their professional duties.^23 24^ Medical professionals could feel uncertain about how to balance these roles,^12 16 25^ a problem known as ‘dual agency’.^26^ Along with that, HA conceptualisations have shifted over time from a collective profession-wide responsibility to an individual physician’s role,^27^ with recent perspectives advocating for a focus on planetary health.^28^ This shift raises questions about which aspects of HA should be addressed at a profession-wide level, which fall under the responsibility of an individual physician, and which are the responsibility of society and its citizens.^20 29^

The evolving diversity in HA conceptualisations highlights the need for an in-depth understanding of the HA role within medical education. A clearer understanding of HA conceptualisations will facilitate a shared narrative that fosters the foundation of an educational environment where HA competencies can reach their full potential. Currently, a comprehensive analysis of HA role conceptualisations across the continuum of medical education is lacking in the literature. To address this gap, the primary purpose of this scoping review is to clarify conceptual dimensions of HA competence in the literature as applied to medical education. In this paper, we used the term ‘health advocacy’ to refer to all the conceptual dimensions that compose it.

### Review questions

What is known about the concept of HA competence in medical education? This question is broken down into subquestions:

- What is the nature and extent of the scientific literature on the HA role in medical education?
- What are the conceptual dimensions and boundaries of the HA role in medical education?
- How do these conceptualisations differ over each phase of medical education?
- How do these conceptualisations differ over time?

We decided on a scoping review since it is well-suited for bringing together and reporting on heterogeneous conceptualisations in literature.^30^ A preliminary search of Embase, MEDLINE, Cochrane Library and Google Scholar was conducted. No comprehensive review of HA conceptualisations over the whole scope of medical education was identified. By viewing the perspectives side by side, this review will lay the groundwork for future studies, whether through a consensus-building process towards a shared understanding of HA in medical education or a focused analysis of HA in specific education contexts or settings. We further propose that identifying points of convergence, where agreement on key elements of HA is achieved, will help establish a shared understanding of the role. At the same time, recognising areas of divergence, where agreement may be challenging or impossible in nature, will inform future discussions regarding what should be taught and assessed concerning HA competencies.

## Methods and analysis

### Protocol and registration

The proposed scoping review will follow the JBI (formerly Joanna Briggs Institute) methodology for conducting scoping reviews and adhere to the Preferred Reporting Items for Systematic Reviews and Meta-Analyses (PRISMA) extension for Scoping Reviews checklist.^31^ Additionally, the best practice guidance and reporting items for the development of scoping review protocols^32^ and recommendations for extraction, analysis and presentation of results in scoping reviews^33^ will be used. Our protocol serves as a blueprint for the scoping review and is intended to reduce potential reporting biases. Any changes made to the protocol during the review process will be clearly documented and explained in the scoping review. The protocol was preregistered with the Open Science Framework.

### Eligibility criteria

#### Population

For this review, only articles that focus on students and physicians who receive or provide medical education, for example, medical students/trainees, residents, fellows and practising physicians across all levels of experience, will be eligible. This encompasses physicians in both general practice and specialised medical fields, as well as those engaged in formal educational roles, such as clinical educators. Additionally, articles addressing the medical profession as a whole will also be considered eligible.

#### Concept

The concept under investigation in this scoping review is ‘health advocacy competence’, as defined by the CanMEDS framework.^9^ We carefully selected terms for ‘health advocacy’ and ‘competence’. Eligible terms for HA will include ‘advocacy’, ‘activism’ and ‘agency’ related to ‘health’ if they are the primary study topic or key component of theoretical discussions on competence. However, broader terms like ‘involvement’, ‘engagement’, ‘responsibility’, ‘accountability’, ‘health promotion’, ‘system change’ and ‘health equity’ will not be eligible unless the authors explicitly link such terms to HA. We have predetermined that we will not infer it on the authors’ behalf. Eligible terms for ‘competence’ will include all activities that relate to attainment, retention and/or loss of knowledge, skills and attitudes, such as ‘life-long learning’ and ‘skill’.

#### Context

This review focuses on HA in the medical education context, as defined, following the definition of the World Medical Association: ‘medical education consists of basic medical education, postgraduate medical education and continuing professional development. Medical education is a dynamic process that commences at the start of basic medical education (medical school) and continues until a physician retires from active practice’.^34^ The different phases of medical education, such as undergraduate, graduate, postgraduate and lifelong/continuous learning, will be eligible. Additionally, articles addressing the medical curriculum as a whole will be considered eligible. We excluded the term ‘foundation’ due to its multiple meanings beyond the UK-specific postgraduate ‘F1 and F2’ years. No restrictions were placed on publication date, geography or language, as we aim for a comprehensive understanding of HA conceptualisations over borders of countries, cultures, traditions and healthcare systems.

#### Types of information sources

Eligible sources include theoretical (eg, reviews) and empirical studies (qualitative, quantitative or mixed-methods), as well as opinion-based articles such as perspectives and editorials.

Conference abstracts and notes/comments were excluded to manage the overall number of results. Additionally, most conference abstracts are eventually published in full within the medical education and practice literature, providing further justification for their exclusion.

#### Search strategy

The search strategy was developed through a collaborative effort between an experienced information specialist (MFME) and the lead author (WRWO) and revised by the other team members. The search was developed in embase.com, optimised for sensitivity and then translated to other databases using the method as described by Bramer *et al*.^35^ The search contained terms for (1) ‘health advocacy’, (2) ‘competence’ and (3) ‘medical education’. Relevant terms from titles, abstracts and index terms were explored and refined through discussions between the research team and, once agreed on, were incorporated into the search. Terms were combined with Boolean operators (AND, OR), and proximity operators were used to combine terms into phrases. The search was carried out in eight electronic databases on 29 October 2024: Embase, MEDLINE, Cochrane Library, Web of Science, CINAHL, ERIC, PsycINFO and LILACS. Additionally, a search was performed in Google Scholar, from which the 100 highest-ranked references were downloaded using the software Publish or Perish, V.8.0. The full search strategies of all databases are available in online supplemental Material 1 and will be updated prior to the submission of the scoping review to ensure all data are current using the methods as described by Bramer and Bain.^36^ The search strategies for MEDLINE and Embase used relevant thesaurus terms from the Medical Subject Headings (MeSH) and Emtree, respectively. In all databases, terms were searched in titles, abstracts and author keywords. The searches in Embase, MEDLINE, Web of Science, ERIC, CINAHL and PsycINFO were limited to exclude conference abstracts and notes/comments. We did not browse unindexed journals in the field. The references were imported into EndNote, and duplicates were removed by MFME using the method described by Bramer *et al*.^37^ Additionally, Covidence identified duplicates that had been inadvertently missed.

#### Data management

The web-based collaboration platform, Covidence (2025), will be used to screen articles and track citations to search for additional articles. Erasmus MC’s internal research project management system will be used for study registration and archiving. Zotero V.7.0 is used to manage references.

#### Selection process

Two reviewers (WRWO and LTvP) will independently apply the inclusion criteria for title and abstract screening. To facilitate calibration, the first meeting will focus on creating a shared understanding of the inclusion criteria. A pilot test on the first hundred articles will be performed and repeated until 90% agreement is reached. Title and abstract screening will be followed by full-text reading of the included articles. If an article does not meet the inclusion criteria after full-text reading, it will be excluded if both reviewers agree. If disagreements arise or if adjustments to the iteratively developed exclusion criteria are needed during the different stages of the screening process, a third and fourth team member (DC-F. and SJO) will be consulted. For articles written in languages other than those that can be translated by our team (eg, Dutch, English, Spanish and Portuguese), the article will be forwarded to a colleague who is a native speaker of the original language. If a native speaker is not available, Erasmus University’s internal AI chatbot will be used. The translation will be critically reviewed, and if any issues remain, the article will be excluded. Any changes to inclusion/exclusion criteria or steps taken to resolve inconsistencies will be thoroughly documented. Excluded sources with the reason for exclusion will be reported at the full-text level and included in the appendices of the scoping review. As the last step, the reference lists of retrieved non-included relevant review articles and of the included references, as well as articles citing these papers, will be scanned for relevant references missed by the search using the methods described by Bramer.^38^ The selection process will be presented in both a narrative and PRISMA flow diagram format (see online supplemental Material 2).^39^

#### Data extraction

To answer the research questions, two researchers will extract the following: Digital Object Identifier; authors; e-mail address of corresponding author; year of publication; country of origin; funding source; title of publication; content/topic(s); study objective(s)/aim(s); type of evidence source; study design; study population; language of data; main outcome; conclusion; future directions; conceptualisations and practical applications. Our study predefined various forms of HA conceptualisations in medical education, including definitions, frameworks, models, theories and conceptual metaphors (for detailed information, see data guidance sheet on online supplemental Material 3). Practical applications stem from underlying conceptualisations and serve as their reflections, but they are distinct from conceptualisations themselves. Therefore, we extract practical applications (eg, examples that demonstrate HA in action) separately. A data charting template, based on the guidance sheet, was developed to support the authors in systematically extracting and comparing the results. To minimise researcher bias and ensure the reliability and feasibility of the template, a pilot test will be conducted by two members of the team (WRWO and LTvP), with iterations made until 90% agreement is reached. Any discrepancies will be addressed during regular meetings with the third and fourth team members (DC-F and SJO). Following the pilot phase, data extraction will proceed sequentially by each reviewer, with regular checks for consistency. The data extraction process will be iterative, allowing for updates to the guidance sheet and charting form as the study progresses. If necessary, authors of the original studies may be contacted to request missing or supplementary data. Any deviations from the protocol will be carefully documented in the scoping review.

#### Data analysis and presentation

The scoping review will be written in English. A descriptive-analytical approach will be used for summarising the data. Quantitative data will include a descriptive numerical summary of the study characteristics and frequency counts of data extraction items. A basic qualitative content analysis will be conducted using inductive open coding to group the different HA conceptualisations into overarching categories, following Pollock *et al*.^33^ Then, the range of general codes will be presented over time, categorised into four periods: before 1996 the (the period before the year in which HA was formally defined within the CanMEDS framework in 1996),^3^ 1996–2010, 2010–2020 and 2020–current. These timeframes may be adjusted as we analyse the data, allowing the findings to guide our final decisions. Subsequently, the range of general codes will be presented for each phase of medical education, using the four pillars: preclinical, clinical, postgraduate and continual professional development. Data will be presented visually in figures whenever possible, with consideration to colour blindness using Colour Oracle V.1.3.

### Ethics and dissemination

This scoping review does not involve any human participants. Consequently, there is no need for ethical approval. The dissemination of the findings will take place through the submission of a single manuscript to a high-quality peer-reviewed journal and will be disseminated through scientific literature and presentations at key conferences.

## Supplementary material

10.1136/bmjopen-2024-097894 online supplemental file 1

10.1136/bmjopen-2024-097894 online supplemental file 2

10.1136/bmjopen-2024-097894 online supplemental file 3

## References

[1] Earnest MA, Wong SL, Federico SG (2010). Perspective: Physician Advocacy: What Is It and How Do We Do It?. Acad Med.

[2] Parkes MW, Poland B, Allison S (2020). Preparing for the future of public health: ecological determinants of health and the call for an eco-social approach to public health education. Can J Public Health.

[3] Frank JR (2004). A history of canmeds – chapter from royal college of physicians of canada 75th anniversary history.

[4] Doobay-Persaud A, Adler MD, Bartell TR (2019). Teaching the Social Determinants of Health in Undergraduate Medical Education: a Scoping Review. J Gen Intern Med.

[5] Agrawal N, Lucier J, Ogawa R (2023). Advocacy Curricula in Graduate Medical Education: an Updated Systematic Review from 2017 to 2022. J Gen Intern Med.

[6] McDonald M, Lavelle C, Wen M (2019). The state of health advocacy training in postgraduate medical education: a scoping review. Med Educ (Chicago Ill).

[7] Scott MD, McQueen S, Richardson L (2020). Teaching Health Advocacy: A Systematic Review of Educational Interventions for Postgraduate Medical Trainees. Acad Med.

[8] Mihan A, Muldoon L, Leider H (2022). Social accountability in undergraduate medical education: A narrative review. *Educ Health*.

[9] Frank JR, Snell LS, Sherbino J (2015). CanMeds 2015 Physician Competency Framework.

[10] LaDonna KA, Kahlke R, Scott I (2023). Grappling with key questions about assessment of the Health Advocate role. Can Med Ed J.

[11] Dobson S, Voyer S, Hubinette M (2015). From the clinic to the community: the activities and abilities of effective health advocates. Acad Med J Assoc Am Med Coll.

[12] LaDonna KA, Watling CJ, Cristancho SM (2021). Exploring patients’ and physicians’ perspectives about competent health advocacy. Med Educ (Chicago Ill).

[13] Hubinette MM, Scott I, van der Goes T (2021). Learner conceptions of health advocacy: ‘Going above & beyond’ or ‘kind of an expectation’. Med Educ (Chicago Ill).

[14] de Bok FE, Hermans J, Duvivier RJ (2024). Conceptualization and teaching health advocacy in undergraduate medical education: a document analysis. BMC Med Educ.

[15] Farrer L, Marinetti C, Cavaco YK (2015). Advocacy for Health Equity: A Synthesis Review. Milbank Quarterly.

[16] Hart E, Kuijpers G, Laverack G (2021). The Process Leading to Physician Activism for Sustainable Change. Sustainability.

[17] Mu L, Shroff F, Dharamsi S (2011). Inspiring Health Advocacy in Family Medicine: A Qualitative Study. *Educ Health*.

[18] Kahlke R, Scott I, van der Goes T (2023). Health advocacy among medical learners: Unpacking contextual barriers and affordances. Med Educ (Chicago Ill).

[19] Scott I, Hubinette M, Van der Goes T (2024). Through a Tainted Lens: A Qualitatve Study of Medical Learners’ Thinking About Patient ‘Deservingness’ of Health Advocacy. Perspect Med Educ.

[20] Dharamsi S, Ho A, Spadafora SM (2011). The Physician as Health Advocate: Translating the Quest for Social Responsibility Into Medical Education and Practice. Acad Med.

[21] Endres K, Burm S, Weiman D (2022). Navigating the uncertainty of health advocacy teaching and evaluation from the trainee’s perspective. Med Teach.

[22] Law M, Leung P, Veinot P (2016). A Qualitative Study of the Experiences and Factors That Led Physicians to Be Lifelong Health Advocates. Acad Med.

[23] Kanter SL (2011). On Physician Advocacy. Acad Med.

[24] Dobson S, Voyer S, Regehr G (2012). Perspective: agency and activism: rethinking health advocacy in the medical profession. Acad Med J Assoc Am Med Coll.

[25] Burm S, Cristancho S, Watling CJ (2023). Expanding the advocacy lens: using photo-elicitation to capture patients’ and physicians’ perspectives about health advocacy. Adv in Health Sci Educ.

[26] Tilburt JC (2014). Addressing Dual Agency: Getting Specific About the Expectations of Professionalism. Am J Bioeth.

[27] Hubinette M, Dobson S, Towle A (2014). Shifts in the interpretation of health advocacy: a textual analysis. Med Educ.

[28] Green S, Labine N, Luo OD (2023). Planetary Health in CanMEDS 2025. Can Med Ed J.

[29] Welie JVM (2012). Social contract theory as a foundation of the social responsibilities of health professionals. Med Health Care and Philos.

[30] Munn Z, Peters MDJ, Stern C (2018). Systematic review or scoping review? Guidance for authors when choosing between a systematic or scoping review approach. BMC Med Res Methodol.

[31] Peters MD, Godfrey C, McInerney P, Aromataris E, Lockwood C, Porritt K (2020). JBI manual for evidence synthesis.

[32] Peters MDJ, Godfrey C, McInerney P (2022). Best practice guidance and reporting items for the development of scoping review protocols. JBI Evidence Synthesis.

[33] Pollock D, Peters MDJ, Khalil H (2023). Recommendations for the extraction, analysis, and presentation of results in scoping reviews. JBI Evidence Synthesis.

[34] World Medical Association WMA statement on medical education. https://www.wma.net/policies-post/wma-statement-on-medical-education.

[35] Bramer WM, De Jonge GB, Rethlefsen ML (2018). A systematic approach to searching: an efficient and complete method to develop literature searches. jmla.

[36] Bramer W, Bain P (2017). Updating search strategies for systematic reviews using EndNote. *jmla*.

[37] Bramer WM, Giustini D, de Jonge GB (2016). De-duplication of database search results for systematic reviews in EndNote. *J Med Libr Assoc*.

[38] Bramer WM (2018). Reference checking for systematic reviews using Endnote. *jmla*.

[39] Page MJ, McKenzie JE, Bossuyt PM (2021). The PRISMA 2020 statement: an updated guideline for reporting systematic reviews. BMJ.

