# Supplementary material for: Bringing together conceptualisations of the health advocacy competence across the continuum of medical education: a scoping review protocol
Source: BMJ Open. 2025 Jul 25;15(7):e097894. doi: 10.1136/bmjopen-2024-097894 (PMC12306260; doi:10.1136/bmjopen-2024-097894)
Supplement: Supplementary file 4 [file bmjopen-2024-097894.reviewer_comments.pdf]

PEER REVIEW HISTORY

BMJ Open publishes all reviews undertaken for accepted manuscripts. Reviewers are asked to complete a checklist review form and are provided with free text boxes to elaborate on their assessment. These free text comments are reproduced below.

ARTICLE DETAILS

Title (Provisional)

Bringing conceptualizations of the health advocacy competence across the continuum of medical education together: a scoping review protocol

Authors

Oosthoek, Wilma R.W.; Cecilio-Fernandes, Dario; Engel, Maarten F. M.; van Prooijen, Lars T.; Otto, Suzie J.; Woltman, Andrea M

VERSION 1 - REVIEW

**Reviewer**	**1**
**Name**	**Jalali, Katayoun**
**Affiliation**	**Shiraz University of Medical Sciences, Medical Education**
**Date**	**27-Jun-2025**
**COI**	**None**

With thanks to the respected researchers

I doubt about Mesh terms used, particularly the term 'health protection.' Please Revise accordingly.

**Reviewer**	**2**
**Name**	**Harrison, David**
**Affiliation**	**University College London, Research Department for Medical Education**
**Date**	**27-Jun-2025**
**COI**	**None**

I would like to particularly commend the authors for their thought and consideration of inclusivity and accessibility to a diversity of readers.

While this scoping review protocol is designed to build on previous reviews of Health Advocacy, it is welcome and timely that the review focuses on establishing through analysis

a consistent conceptualization of HA. With continuing debate around various HA roles and where boundaries are blurred and contested, it is important to clarify the concepts while also allowing for flexibility in a role that needs to be both professional and personalised.

The PRISMA-ScR methodology has been appropriately applied and their is transparency of methodology throughout the paper, so achieving the aim of reducing potential bias. Something that is worth highlighting is the inclusiveness around language as part of the review process. Moving beyond the multi-lingual proficiencies of the review team, there is appropriate use made of AI for translation. So many reviews are restricted to purely English papers so this is a very welcome aspect of the review protocol. However, I would urge the authors to engage in checks on any online translations and to seek out assurances on the validity of AI translation and on how any data from this scoping review process may be used for AI training.

Overall, I commend the authors on creating an inclusive protocol that clearly addresses gaps in the current literature and will also advance the debate around conceptualisation of Health Advocacy and how the healthcare sector perceives the role of professional health advocacy.

VERSION 1 - AUTHOR RESPONSE

Reviewer 1
Dr. Katayoun Jalali, Shiraz University of Medical Sciences

Comments to the Author:
Comment 1: With thanks to the respected researchers, I doubt about Mesh terms used, particularly the term 'health protection.' Please Revise accordingly.
Response: Thank you for raising this point regarding the MeSH terms used in our search strategy. From your comment, it was unclear whether the suggestion was to include or exclude the term “health protection”. We reviewed our search strategy thoroughly and can confirm that the term “health protection” does not appear in it (see Supplemental Material 1). Additionally, we note that “health protection” is not a recognized MeSH term.

If the intention was to recommend inclusion of this term as a search term, we appreciate the suggestion. We would like to clarify that our search strategy was developed in collaboration with a medical librarian and based on the methodology described by Bramer et al. (2018), as referenced in the Methods section. This approach ensures the optimal use and translation of terms across databases.

Should you feel that a specific concept related to “health protection” is insufficiently covered, we would welcome further clarification.

Bramer WM, Jonge GB de, Rethlefsen ML, Mast F, Kleijnen J. A systematic approach to searching: an efficient and complete method to develop literature searches. J Med Libr Assoc. 2018 Oct 4;106(4):531–41.

Reviewer 2
Dr. David Harrison, University College London

Comments to the Author:
Comment 1: I would like to particularly commend the authors for their thought and consideration of inclusivity and accessibility to a diversity of readers. While this scoping review protocol is designed to build on previous reviews of Health Advocacy, it is welcome and timely that the review focuses on establishing through analysis a consistent conceptualization of HA. With continuing debate around various HA roles and where boundaries are blurred and contested, it is important to clarify the concepts while also allowing for flexibility in a role that needs to be both professional and personalised.
Response: Thank you for sharing your thoughtful feedback and personal reflections on HA competence**.** We look forward to approaching the various viewpoints and interpretations with sensitivity, to give visibility to both frequently heard and less commonly heard voices in our scoping review.

Comment 2: The PRISMA-ScR methodology has been appropriately applied and their is transparency of methodology throughout the paper, so achieving the aim of reducing potential bias.
Response: Thank you for your compliment.

Comment 3: Something that is worth highlighting is the inclusiveness around language as part of the review process. Moving beyond the multi-lingual proficiencies of the review team, there is appropriate use made of AI for translation. So many reviews are restricted to purely English papers so this is a very welcome aspect of the review protocol. However, I would urge the authors to engage in checks on any online translations and to seek out assurances on the validity of AI translation and on how any data from this scoping review process may be used for AI training.
Response: We appreciate your recognition of our inclusive language approach, as well as your valuable caution regarding the use of AI in translation and the future use of data.

First, we reflected critically on our use of AI. While AI is increasingly integrated into research workflows, we are aware of the ethical and epistemological concerns it raises in relation to data ownership, privacy, ecological sustainability and the potential for inaccurate outputs (Zhao et al, 2025; Samuel & Ritchie, 2023). In the original version of our protocol, we noted in the "selection process" paragraph that we would consult AI translation tools in cases where our team lacked sufficient language expertise (in Dutch, English, Spanish and Portuguese). Upon reflection, and in line with your advice, we have revised this procedure. In the updated protocol, we now prioritize consulting native speakers first. Only if a native speaker is not available AI will be used, followed by critical team review. In cases of persistent uncertainty, the article will be excluded from the review.

Second, we can confirm that no data from this scoping review protocol or the final review will be used to train AI models. If AI tools are employed, we will adhere with the General Data Protection Regulation (GDPR) and AI guidelines of our institute, using only our internal AI chatbot to ensure that data from closed access journals remains protected. As we plan to publish open access, we acknowledge that we cannot fully control how the scoping review content may be used beyond our own sphere of responsibility. We will remain transparent about this limitation and will stay closely engaged with colleagues from the Medical Ethics, Philosophy & Medical History department, who are developing guidelines for the ethical and responsible use of AI.

Zhao X, Yang X, Zhang M, Sun W. The Absence of Metaphor in AI-Generated Offensive Language: Implications for Conceptual Metaphor Theory and AI Ethics. Int J Educ Humanit. 2025 Jun 28;5(3):590–601.

Samuel G, Richie C. Reimagining research ethics to include environmental sustainability: a principled approach, including a case study of data-driven health research. J Med Ethics. 2023 Jun;49(6):428–33.

Comment 4: Overall, I commend the authors on creating an inclusive protocol that clearly addresses gaps in the current literature and will also advance the debate around conceptualisation of Health Advocacy and how the healthcare sector perceives the role of professional health advocacy.
Response: Thank you.
